# Cytotoxic and Senolytic Effects of Methadone in Combination with Temozolomide in Glioblastoma Cells

**DOI:** 10.3390/ijms21197006

**Published:** 2020-09-23

**Authors:** Bernd Kaina, Lea Beltzig, Andrea Piee-Staffa, Bodo Haas

**Affiliations:** 1 Institute of Toxicology, University Medical Center, Obere Zahlbacher Str. 67, 55131 Mainz, Germany; lea.beltzig@uni-mainz.de (L.B.); pieean01@uni-mainz.de (A.P.-S.); 2 Federal Institute for Drugs and Medical Devices, Kurt-Georg-Kiesinger-Allee 3, 53175 Bonn, Germany; bodo.haas@bfarm.de

**Keywords:** glioblastoma, methadone, temozolomide, apoptosis, drug resistance, cancer therapy, senescence

## Abstract

Methadone is an analgesic drug used for pain treatment and heroin substitution. Recently, methadone has been proposed to be useful also for cancer therapy, including glioblastoma multiforme (GBM), the most severe form of brain cancer, because experiments on cultured glioma cells treated with doxorubicin showed promising results. Doxorubicin, however, is not used first-line in GBM therapy. Therefore, we analyzed the cytotoxic effect of methadone alone and in combination with temozolomide, a DNA-alkylating drug that is first-line used in GBM treatment, utilizing GBM-derived cell lines and a human fibroblast cell line. We show that methadone is cytotoxic on its own, inducing apoptosis and necrosis, which was observed at a concentration above 20 µg/mL. Methadone was similar toxic in isogenic MGMT expressing and non-expressing cells, and in LN229 glioblastoma and VH10T human fibroblasts. The apoptosis-inducing activity of methadone is not bound on the opioid receptor (OR), since naloxone, a competitive inhibitor of OR, did not attenuate methadone-induced apoptosis/necrosis. Administrating methadone and temozolomide together, temozolomide had no impact on methadone-induced apoptosis (which occurred 3 days after treatment), while temozolomide-induced apoptosis (which occurred 5 days after treatment) was unaffected at low (non-toxic) methadone concentration (5 µg/mL), and at high (toxic) methadone concentration (20 µg/mL) the cytotoxic effects of methadone and temozolomide were additive. Methadone is not genotoxic, as revealed by comet and γH2AX assay, and did not ameliorate the genotoxic effect of temozolomide. Further, methadone did not induce cellular senescence and had no effect on temozolomide-induced senescence. Although methadone was toxic on senescent cells, it cannot be considered a senolytic drug since cytotoxicity was not specific for senescent cells. Finally, we show that methadone had no impact on the MGMT promoter methylation. Overall, the data show that methadone on glioblastoma cells in vitro is cytotoxic and induces apoptosis/necrosis at doses that are above the level that can be achieved in vivo. It is not genotoxic, and does not ameliorate the cell killing or the senescence-inducing effect of temozolomide (no synergistic effect), indicating it has no impact on temozolomide-induced signaling pathways. The data do not support the notion that concomitant methadone treatment supports temozolomide-based chemotherapy.

## 1. Introduction

Glioblastoma (WHO grade IV, glioblastoma multiforme, GBM) accounting for ~70% of high- grade malignant gliomas, have a dismal prognosis. The median overall survival time of newly diagnosed GBM is maximally 14 months, and the median overall 2-year survival rate is 26%, with a wide range of outcomes [1]. An important prognostic marker is the DNA repair protein *O*^6^-methylguanine-DNA methyltransferase (MGMT), and patients harboring the methylated (silenced) MGMT promoter have a better prognosis than patients with the tumor-unmethylated (active) MGMT promoter, with overall survival times of 12.2 and 18.2 months, respectively [2]. Standard therapy includes resection of the tumor with maximum safe margin, followed by radiotherapy and concomitant chemotherapy with temozolomide (TMZ) [3]. Taken the bad prognosis of patients suffering from glioblastoma, it is obvious that there is an urgent need for supporting therapeutic strategies.

It has been reported that d,l-methadone (MTD), an opioid agonist used for heroin substitution and a widely used analgesic in pain therapy, induces cell death and supports the cytotoxic effect of doxorubicin in leukemia cells [4]. Extending this work, the same group studied MTD on glioblastoma cell lines and reported that the opioid is able to enhance the apoptosis-inducing effect of doxorubicin in this cell system [5]. This was explained by assuming that MTD impacts doxorubicin uptake and attenuates its efflux, which would enhance the intracellular level of doxorubicin and, consequently, its cytotoxic activity [5]. Based on this work, a trial has been initiated treating glioma patients (grade II–IV) with MTD, which occurred independent of their neuro-oncological treatment [6]. In view of the patient heterogeneity, small group size and lacking stratification of the control group, a conclusion as to the therapeutic benefit of MTD in mono- or combined therapy could not be drawn.

Although a beneficial effect of MTD in glioblastoma therapy cannot per se be neglected, it is important to note that glioblastoma is not treated first-line with the topoisomerase II-inhibitor doxorubicin, which has been used in previous studies. First-line chemotherapeutics for glioma (grade III and IV) is the DNA alkylating agent temozolomide (TMZ) [3] and, therefore, it is desirable to study the interaction of MTD with TMZ. This genotoxic anticancer drug is a triazene derivative that does not need metabolic activation and methylates DNA at various sites [7]. One of the methylation products, O^6^-methylguanine, is the preponderant killing lesion, inducing apoptosis, autophagy, and senescence [8]. The survival pathways, including DNA repair by MGMT [7,9], and death pathways activated upon TMZ treatment were described in detail [8,9,10]. Cell death is based on DNA mismatch repair-mediated processing of *O*^6^-methylguanine/thymine mispairs in the DNA, formation of DNA double-strand breaks (DSB), activation of the DNA damage response and downstream cell death pathways. Here, we set out to determine whether MTD has an impact on the well-known cytotoxic effect of TMZ on glioblastoma cells in an in vitro setting. Our data do not support the hypothesis that MTD has a synergistic effect on glioblastoma cells, ameliorating apoptosis, necrosis, and cellular senescence following TMZ treatment, although MTD itself, at high dose levels (>20 µg/mL), was apoptosis/necrosis-inducing, irrespective of the MGMT status of glioblastoma cells. We also report that MTD does not induce cellular senescence, is not genotoxic, and has no impact on epigenetic MGMT regulation.

## 2. Results

### 2.1. Viability Assays

**Figure 1:**
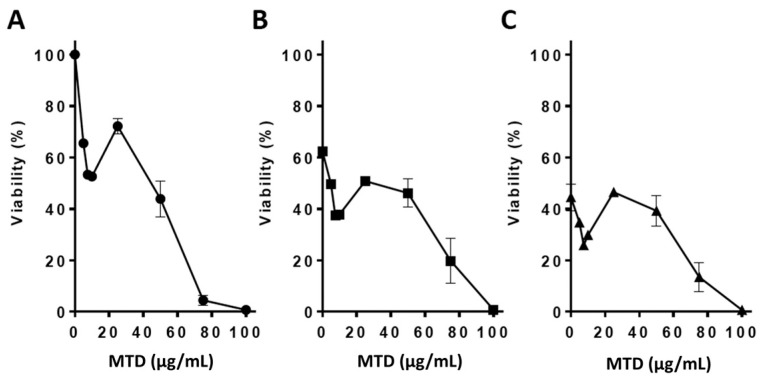
Cytotoxicity of methadone (MTD) on LN229 cells without and with co-treatment with temozolomide (TMZ). (**A**) Viability of LN229 cells, measured by the MTT assay, as a function of MTD concentration in the medium. (**B**) Viability of LN229 cells treated with 20 µM TMZ and (**C**) 75 µM TMZ and increasing concentrations of MTD. The MTT assay was performed 96 h after the addition of MTD to the medium. Data are the mean of three independent experiments.

First, in dose-finding experiments, we set out to determine whether MTD is cytotoxic in LN229 glioblastoma cells. As shown in Figure 1A, MTD reduced the viability of LN229 cells dose dependently. Already with a concentration of 5 µg/mL, viability was significantly reduced (by ~30%), and with 100 µg/mL, all cells in the population died. The data shows that MTD itself reduces the viability of GBM cells.

Next, we addressed the possibility that MTD exerts a supportive effect on the viability brought about by TMZ in LN229 cells. Treatment of exponentially growing LN229 cells occurred first with TMZ and, 2 h later, by the addition of MTD to the medium. TMZ treatment with a dose of 20 and 75 µM reduced the viability by about 40 and 60%, respectively, and MTD further reduced the viability (Figure 1B,C).

**Figure 2:**
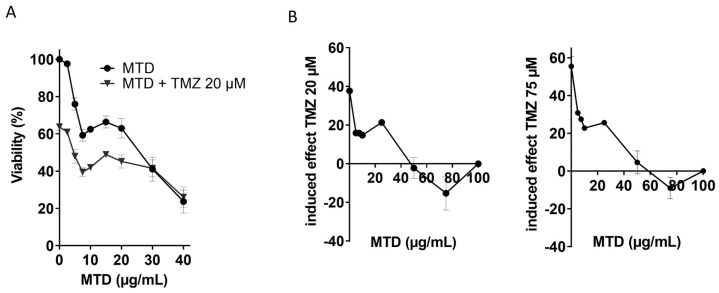
Cytotoxicity of TMZ measured in the MTT assay in the absence and presence of MTD. (**A**) Exponentially growing cells were treated with TMZ and increasing concentrations of MTD. Data are the mean of three separate experiments. (**B**) Induced effect of TMZ (20 and 75 µM, left and right panel, respectively) on the viability of LN229 cells co-treated with MTD. The toxic effect induced by MTD was subtracted from the effect obtained after combined treatment, shown in panel A for 20 µM TMZ. Viability was measured 120 h after addition of the drugs to the medium.

In another experimental series, we measured the viability in a dose range of up to 40 µg/mL MTD in the absence and presence of TMZ. In this case, cells were treated simultaneously with both drugs. The viability curves displayed a nearly parallel course up to a dose level of 20 µg/mL MTD, indicating an additive mode of action. At high MTD concentrations (>20 µg/mL), TMZ seems to be even less effective than expected (Figure 2A), which was also observed in other experiments (see below). We should note that the viability curves for MTD have a biphasic shape with an initial nadir at 7.5 µg/mL, which was observed in all cell viability assays performed. In the viability assays, inhibition of proliferation and cell death reduce MTT metabolic activity, which might explain the first nadir, resulting from proliferation inhibition.

The effect of MTD on TMZ-induced cell death was further assessed by subtracting the cytotoxic effect of MTD from the effect observed in the combination treatment, which represents the effect induced by TMZ. The cytotoxic effect of TMZ was completely abrogated if cells were co-treated with MTD with a concentration >40 µg/mL (Figure 2B). This is likely a result of proliferation inhibition since TMZ requires cell cycle progression for the conversion of critical DNA adducts into toxic lesions [11].

### 2.2. Apoptosis and Necrosis Induced by MTD

**Figure 3:**
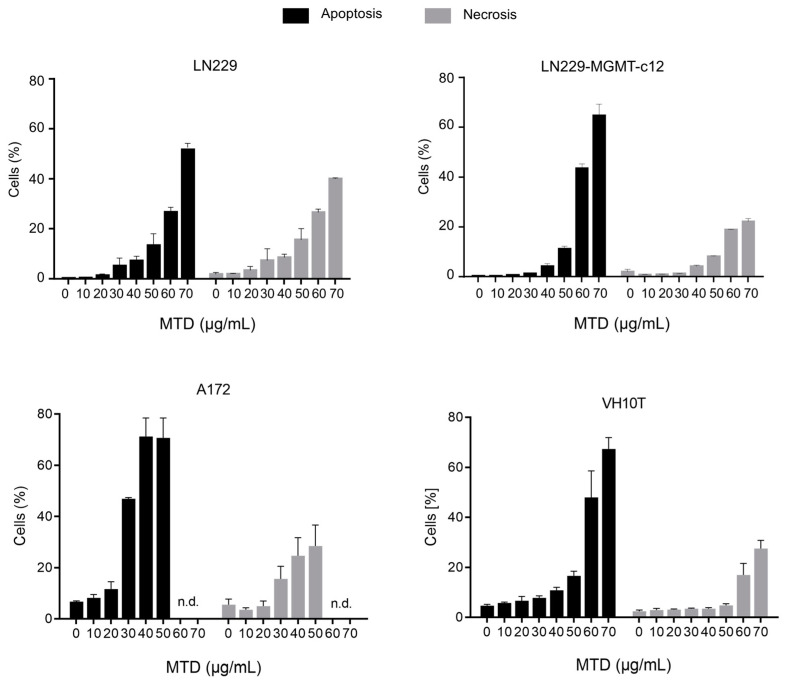
Apoptosis and necrosis induced in different glioblastoma cell lines and human fibroblasts VH10T. Exponentially growing cells were cultivated in medium with increasing concentrations of MTD, harvested, annexin V and propidium iodine (annexin V/PI) stained and measured by flow cytometry. Cells were analyzed 72 h after addition of MTD to the medium. Data are the mean of three independent experiments. n.d., not detectable.

The cytotoxic effect of MTD on glioblastoma cells was substantiated by measuring apoptosis and necrosis (annexin V and propidium iodine (AV/PI) flow cytometry). As shown in Figure 3, apoptosis and necrosis increased concentration-dependently. Glioblastoma A172 cells were clearly more sensitive than LN229. For comparison, we included human diploid fibroblasts, the line VH10T [11], which also showed concentration-dependent apoptosis. The fibroblasts (MGMT proficient) displayed a response similar to LN229 (MGMT deficient), while A172 was more sensitive, showing significant apoptosis/necrosis at a concentration >20 µg/mL MTD. Interestingly, MTD was also effective in inducing apoptosis and necrosis in LN229 cells expressing the DNA repair protein MGMT (LN229-MGMT-c12), indicating that the cytotoxic effect of MTD is independent on O^6^-DNA alkylation lesions.

### 2.3. Is MTD a Senescence Inducing Drug?

**Figure 4:**
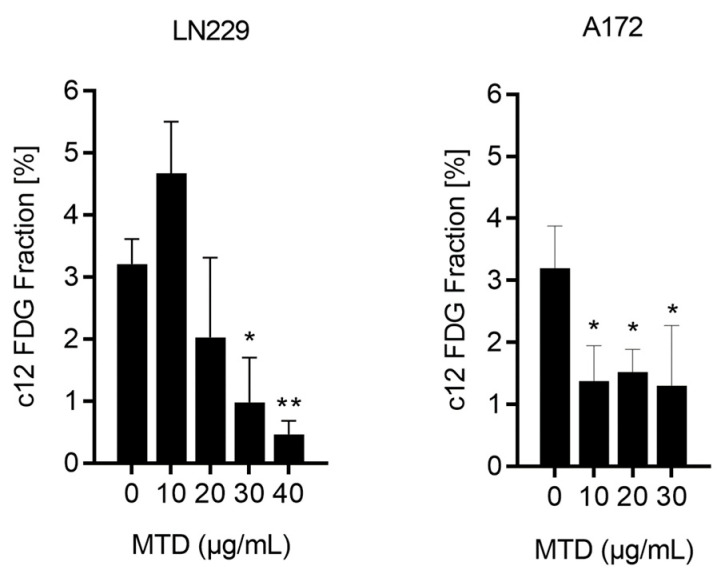
Level of cellular senescence in LN229 and A172 glioblastoma cells treated with MTD. Cells were harvested and measured 72 h after addition of MTD to the medium of exponentially growing cells. Data are the mean of three independent experiments. * *p* < 0.05; ** *p* < 0.01.

Previously, we have shown that glioblastoma cells undergo apoptosis and cellular senescence, if they were treated with DNA damaging agents [8,11]. Therefore, we were interested in assessing whether the cells enter the senescent state upon treatment with MTD. Surprisingly, although cells undergo apoptosis 3 days after the onset of MTD treatment, cellular senescence was not induced under these treatment conditions. The spontaneous level of senescence (as measured by c12FDG flow cytometry) was about 3%, which even declined following treatment, which is likely a result of death of spontaneously occurring senescent cells in the population (Figure 4).

### 2.4. Is the Opioid Receptor Involved?

**Figure 5:**
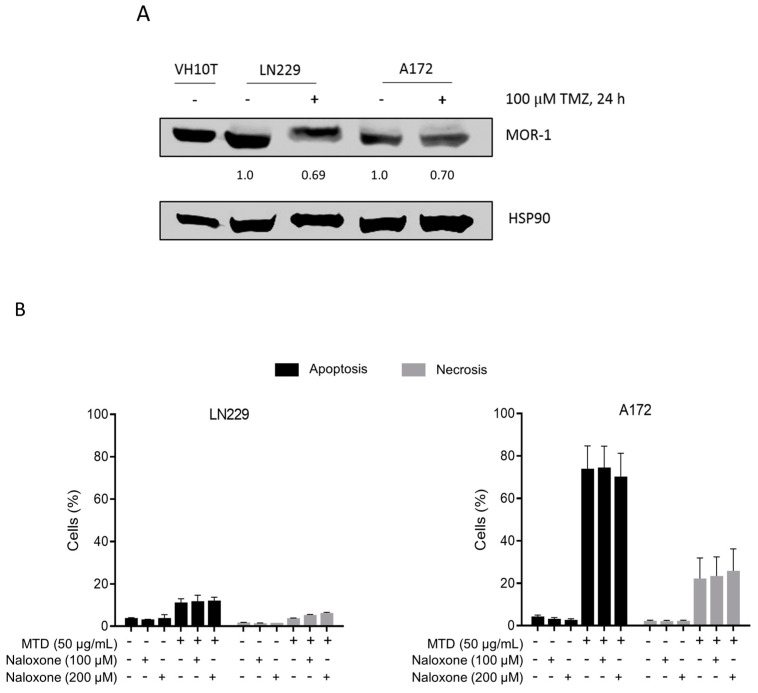
Expression of µ-opioid receptor MOR-1 in LN229 and A172 cells and effect of naloxone. (**A**) For comparison the level in human diploid fibroblasts VH10T are shown. The effect of TMZ on the receptor expression level was quantified in relation to the non-treated control, which was set to 1.0. (**B**) Effect of naloxone on MTD-induced cell death. Naloxone was administered 30 min before the addition of MTD to the medium. Data are the mean of three independent experiments.

LN229 and A172 express the µ-opioid receptor (MOR-1), as human fibroblasts VH10T (used as a control) do (Figure 5A). Interestingly, TMZ treatment reduced the amount of MOR-1, and a lower basal expression level of MOR-1 was observed in A172 cells compared to LN229 (Figure 5A), although this line was more sensitive to apoptosis induction by MTD than LN229 (Figure 3). This may be taken to indicate that the toxic effect of MTD is not mediated by the opioid receptor. This is supported by the finding that naloxone, a competitive inhibitor of opioid receptors, had no effect on MTD-induced apoptosis (Figure 5B). We should note that we used naloxone concentrations that were non-toxic and equimolar to the MTD concentration or even higher (100 and 200 µM). We conclude that cytotoxicity induced by MTD in glioblastoma cells is not mediated by the opioid receptor.

### 2.5. Is MTD-Induced Apoptosis/Necrosis Affected by TMZ?

**Figure 6:**
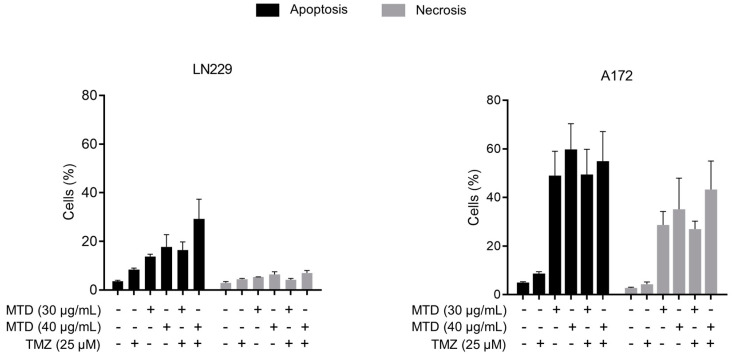
Effect of TMZ on MTD-induced apoptosis/necrosis in LN229 and A172 cells, measured 72 h after treatment. Data are the mean of three independent experiments. The differences between MTD and MTD-TMZ were in both cell lines not significant.

To elucidate whether TMZ has an impact on apoptosis and necrosis induced by MTD, we treated cells with MTD and immediately thereafter with TMZ. As shown in Figure 6, MTD (30 and 40 µg/mL) induced apoptosis/necrosis effectively whereas TMZ (25 µM) was ineffective when measured 3 days after treatment. Co-exposure of MTD and TMZ did not ameliorate the toxic effect of MTD.

### 2.6. Is TMZ-Induced Apoptosis/Necrosis Affected by MTD?

**Figure 7:**
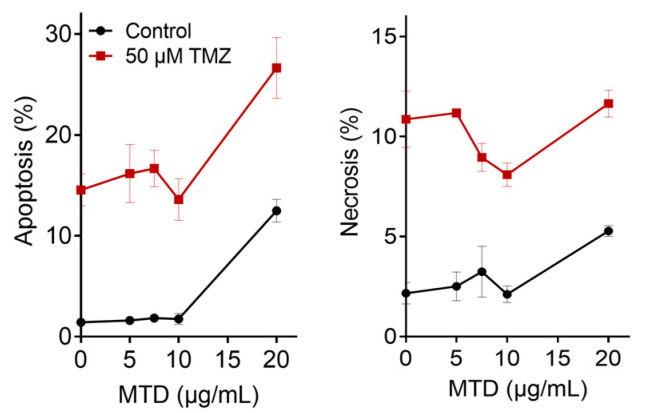
Effect of MTD alone (control) and in combination with TMZ (20 µM) on TMZ-induced apoptosis and necrosis, measured by AV/PI flow cytometry. Cells were harvested and analyzed 96 h after the addition of TMZ and MTD to the medium.

While glioblastoma cells undergo apoptosis/necrosis already 2–3 days after addition of MTD to the medium, the onset of apoptosis following TMZ is much later, starting 4–5 days after treatment [11]. TMZ-induced apoptosis is obviously a late effect, resulting from DNA damage processing and the activation of a complex signaling cascade [12]. To analyze whether MTD has an impact on these processes triggered by O^6^MeG, LN229 cells were treated with TMZ, and 3 h later post-treated with MTD until harvest 96 h later. As shown in Figure 7, MTD was ineffective in inducing apoptosis and necrosis up to a dose of 10 µg/mL. In this dose range, MTD did not enhance the cytotoxic response of TMZ. With a higher concentration of 20 µg/mL, MTD induced apoptosis and ameliorated the effect of TMZ, which was, however, merely additive (Figure 7).

**Figure 8:**
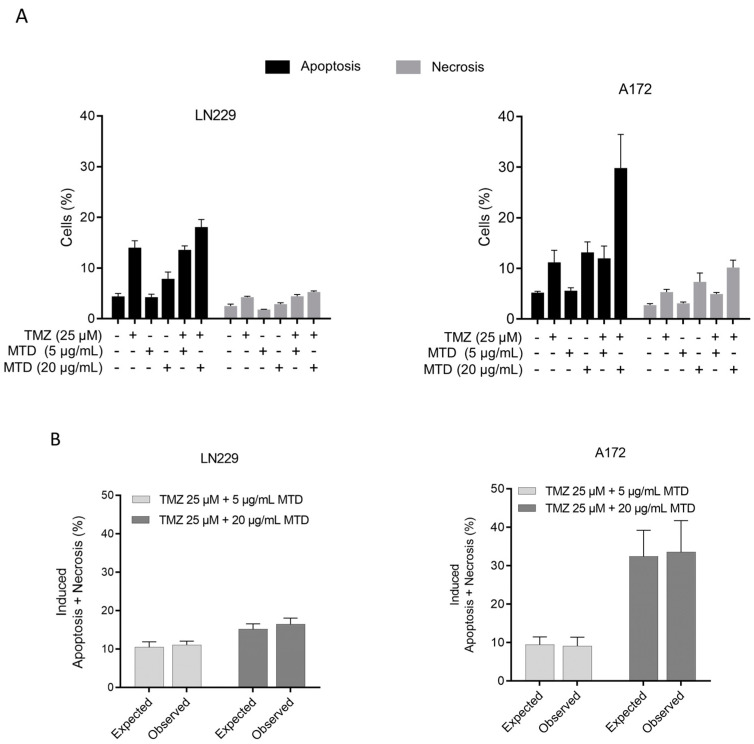
Effect of MTD on TMZ-induced apoptosis and necrosis, measured by AV/PI flow cytometry. (**A**) Percentage of apoptotic and necrotic cells in the population, determined 120 h after treatment. Data are the mean of three independent experiments. (**B**) TMZ-MTD induced cell death (apoptosis/necrosis). Observed levels after combined treatment and expected frequencies on the basis of additivity of single-drug treatment.

In another experimental series, LN229 and A172 cells were treated with TMZ and post-treated with MTD. Again, a subtoxic concentration of MTD (5 µg/mL) had no impact while a cytotoxic MTD concentration (20 µg/mL) ameliorated the apoptotic effect of TMZ (Figure 8A). The measured combined effects and the effects expected on the basis of additivity revealed that cell death induced by TMZ (25 µM) plus a toxic dose of MTD (20 µg/mL) is additive (Figure 8B and Table S1) supporting the data shown above (Figure 7). In conclusion, the data indicate that MTD has no impact on TMZ-induced cell death pathways.

### 2.7. Is TMZ-Induced Senescence Affected by MTD?

**Figure 9:**
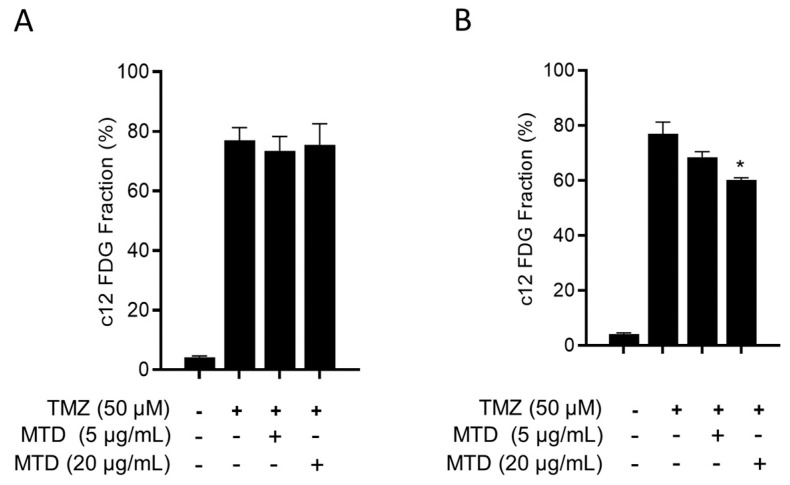
Effect of MTD on TMZ-induced senescence. (**A**) Induction of senescence by TMZ in LN229 cells and effect of MTD co-treatment on senescence induction. (**B**) Effect of MTD on the TMZ-induced senescent cell level. Data are the mean of three independent experiments +/-SEM. * *p* ≤ 0.05.

As outlined above, TMZ is a potent inducer of cellular senescence [8] and, thus, the possibility remained that MTD has an impact on this process. Therefore, we measured the level of TMZ-induced senescence in the absence or presence of MTD. As shown in Figure 9A, 8 days after addition of TMZ to the medium, about 80% of the population was tested positive for the senescence marker c12FDG, and MTD (5 and 20 µg/mL) neither enhanced nor reduced the senescence level. Obviously, MTD has no impact on TMZ-induced senescence pathways.

### 2.8. Is MTD a Senolytic Drug?

Senescent cells can be killed by specific agents through a process called senolysis. To elucidate whether MTD acts as a senolytic drug, we treated the senescent population with MTD and measured the senescent fraction 48h later. As shown in Figure 9B, a subtoxic concentration of MTD had no significant effect on the senescent population while a toxic MTD concentration significantly reduced the senescence level. This, however, was not specific for senescent cells since 20 µg/mL MTD was also toxic for non-senescent (exponentially growing) cells. Obviously, MTD is able to kill senescent and non-senescent cells (see also Figure S1); therefore, it cannot be considered as a senolytic drug, which acts specifically on senescent cells.

### 2.9. Is MTD a DNA Damaging Agent and Does It Impact TMZ-Induced DNA Damage?

**Figure 10:**
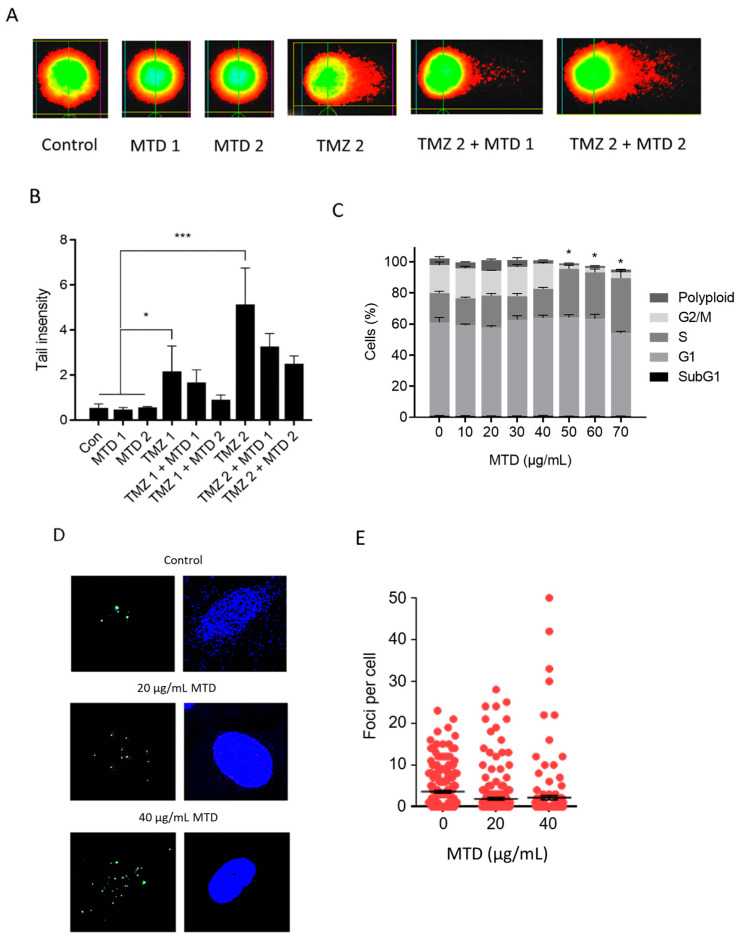
Effect of MTD on genotoxicity of TMZ and cell cycle distribution. (**A**) Comet assay, MTD alone (control) and in combination with TMZ, measured by the alkaline comet assay. Representative pictures are shown. (**B**) Quantification: TMZ 1, 100 µM; TMZ 2, 200 µM; MTD 1, 10 µg/mL, MTD 2, 30 µg/mL. Data represent the mean of three independent experiments ± SEM. * *p* < 0.05; *** *p* < 0.001. (**C**) Cell cycle distribution of LN229 cells treated with increasing concentrations of MTD. * indicates significant difference (*p* > 0.05) of S and G2 fraction from control. (**D**) Representative pictures of γH2AX foci and (**E**) foci levels in LN229 cells measured 24 h after addition of MTD to the medium. The differences in the mean foci number between control and MTD are statistically not significant.

TMZ is a genotoxic anticancer drug that methylates DNA bases in different positions. The N-methylation purines N7-methylguanine, N3-methyladenine, and N3-methylguanine are major alkylation products, and their formation can indirectly be monitored by the alkaline comet assay, which is a sensitive method for the detection of alkali labile and apurinic sites, as well as DNA single-strand breaks. As shown in Figure 10A,B, MTD was not genotoxic in LN229 cells. It also did not ameliorate the genotoxic effect of TMZ. There was even a reduction in tail intensity, which was, however, not statistically significant (Figure 10B). Therefore, MTD appears to have no significant impact on the DNA-damaging capacity of TMZ.

The observed trend of lower tail intensity in the TMZ + MTD treatment schedule could be a result of inhibition of proliferation following MTD treatment since genotoxicity of TMZ is enhanced if cells replicate [12]. Indeed, high concentrations of MTD (>40 µg/mL) significantly enhanced the fraction of S-phase and reduced the fraction of G2-phase cells (Figure 10C). Obviously, at high MTD concentrations, cells do not enter G2 because they became arrested in the S-phase. Therefore, it is reasonable to conclude that cell cycle inhibition upon MTD treatment reduces the genotoxic and toxic effects of TMZ.

A sensitive indicator for DNA damage are γH2AX foci, whereby each focus represents either blocked and collapsed replication forks or free DSBs that trigger the DNA damage response (DDR) [13]. It has further been shown that γH2AX foci are tightly linked to cytotoxic DNA damage [14]. To elucidate whether MTD has the capacity to induce the DDR, we measured the γH2AX level following MTD exposure. There was no significant difference in the mean γH2AX foci level between control and MTD (Figure 10D,E). This supports the notion that MTD, even at cytotoxic concentration, is not genotoxic.

### 2.10. MTD and MGMT Promoter Methylation

**Figure 11:**
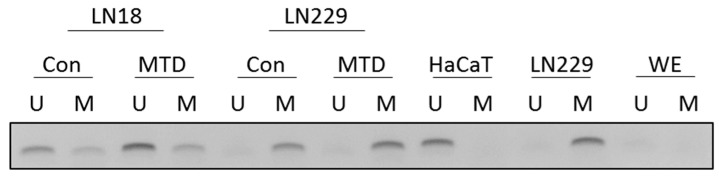
MGMT promoter methylation in glioma cells cultivated in the absence and presence of methadone. Con, control without MTD; MTD, 10 µg/mL for a period of 72 h. Separately harvested extracts of non-treated human HaCaT cells were used as a control for the unmethylated promoter, and LN229 for the methylated promoter. WE, without cell extract. U, unmethylated; M, methylated.

Finally, we considered the possibility that MTD has an impact on MGMT promoter methylation, which is tightly linked to MGMT expression and alkylating drug resistance [15]. Therefore, we measured MGMT promoter methylation by MS-PCR, as described previously [16]. Cultivation of cells in medium containing MTD had no impact on the MGMT promoter methylation status. Thus, neither the unmethylated MGMT promoter of TMZ-resistant LN18 glioblastoma cells was converted into a methylated promoter, nor the methylated promoter of TMZ-sensitive LN229 cells was converted into an unmethylated one (Figure 11). Thus, it appears that MTD has no impact on MGMT promoter methylation.

## 3. Discussion

MTD, an opioid and µ-receptor agonist commonly used for pain treatment of cancer patients, was shown to be cytotoxic first on human leukemia cell lines. Apoptosis was induced in doxorubicin resistant and sensitive lines, which was taken to indicate that the mechanism of cell death is different from the mechanisms leading to doxorubicin resistance [4]. Thereafter, it was reported that MTD-induced cytotoxicity is dependent on the opioid receptor level, regulating doxorubicin influx and efflux [17]. Extending this concept, authors stepped into the glioblastoma cell system and confirmed the findings obtained with leukemia cells, i.e., MTD is cytotoxic and sensitizes glioma cells to doxorubicin [5]. Other anti-cancer drugs were not tested. The findings led to the notion that MTD is useful for GBM therapy and opened a heated debate about the general use of MTD in cancer treatment. Doxorubicin is frequently used in veterinary medicine; thus, the question arose whether MTD is useful also for increasing the effectiveness of therapy of animals. In a recent study with µ-receptor expressing canine cell lines, authors did not observe a potentiation of doxorubicin-induced growth inhibition by MTD and, overall, were unable to confirm the data summarized above [18].

Although doxorubicin is not brain-permeable and not primarily used in glioma therapy (except the pegylated liposomal drug, Caelyx^®®^, used off-label), the findings were taken to indicate that MTD might be useful for monotherapy or as a supportive drug in glioma treatment. Therefore, a first trial was conducted with glioma patients (grade II-IV) that received standard therapy, including TMZ, together with MTD. Although no final conclusions can be drawn from this retrospective study of small group size [6], the regime based on MTD as therapeutic has been widely discussed and propagated in social media. It, thus, became obvious that there is a strong need for more background data on MTD and its impact on TMZ-induced responses.

Aimed at addressing these questions, we conducted a study using the glioblastoma cell lines LN229 and A172 (both are p53 functionally active and MGMT lacking) and a MGMT transfected derivative of LN229 (LN229-MGMT-c12) as well as human fibroblasts (the telomerase immortalized, non-tumorigenic line VH10T). The cells were previously well characterized by us, and others, as to TMZ-induced responses [8,11,19,20]. We observed that MTD is cytotoxic and induces apoptosis and, to a much lesser extent, necrosis in LN229 and A172 cells. A172 was more sensitive than LN229, whereas VH10T showed a response similar to LN229. All cell lines expressed the opioid receptor MOR−1. Interestingly, the expression level did not correlate with their sensitivity to MTD. Experiments with naloxone showed that competitive inhibition of the opioid receptors was without effect on MTD-induced cell death, indicating that opioid receptor-triggered pathways are not involved in MTD-induced cell death.

MTD induces apoptosis/necrosis early (i.e., 2–3 days) after treatment, while TMZ needs a much longer time for lesion processing and activating the cell death pathways (5 to 8 days in glioma cells, dependent on their cell cycle progression [21]). Therefore, we studied in two experimental settings whether MTD and TMZ are interacting. Firstly, we elucidated the effect of TMZ on MTD-induced apoptosis and found that MTD-induced death was not affected by TMZ. Secondly, we elucidated whether MTD has an impact on TMZ-induced apoptosis/necrosis and again, with low (non-toxic) dose of MTD we did not observe drug interaction. At higher (cytotoxic) MTD concentration, the killing effects of MTD and TMZ were additive. The data suggests that the cell death pathways triggered by MTD and TMZ are not converging and involve different mechanisms. We should note that at high MTD concentration (>40 µg/mL) the TMZ-induced decrease in viability (measured in the MTT assay) was even attenuated, presumably due to cell cycle inhibition following MTD, which counteracts TMZ-induced cell death responses [12].

We further show that MTD, even at cytotoxic concentration, was negative in the alkaline comet assay and in the γH2AX foci assay, which strongly indicates that MTD is not a genotoxic drug. This disproves the hypothesis that MTD-induced cytotoxicity is merely a result of oxidative stress generated in MTD exposed cells, which damages the nuclear DNA. We cannot exclude, however, that oxidative stress is provoked in the mitochondria, which causes cell death. Interestingly, we observed equal levels of MTD-induced apoptosis and necrosis in MGMT lacking and MGMT expressing LN229 cells, indicating that MTD induces death irrespective of the MGMT status and thus is able to kill cells that are highly resistant to TMZ and other O^6^-alkylating anticancer drugs, such as nimustine, carmustine, lomustine and others [9]. Cell death induced by these drugs in highly sensitive MGMT lacking cells is a result of activation of complex signaling pathways triggered by the specific DNA lesion O^6^-alkylguanine [10,20,22]. Thus, it can be concluded that the MTD-induced death pathway does not interfere with O^6^-alkylguanine-triggered cell death pathways.

TMZ is not only an apoptosis-inducing agent, it also triggers with high efficiency cellular senescence in glioblastoma cells [8]. The pathways involved were previously described [11]. Having this in mind, we set out to determine whether MTD has an impact on TMZ-induced senescence, which is a late response following TMZ exposure. MTD itself did not induce senescence. We also did not observe an impact of MTD on the capacity of TMZ to induce senescence. This is in line with the notion that the signaling pathways triggered by MTD and TMZ are essentially different. It is important to note that MTD was able to kill LN229 cells arrested in a TMZ-induced senescent state. Agents having this ability are considered senolytic drugs. However, to elicit this response, a concentration of MTD was required that was cytotoxic on proliferating cells and, thus, the effect was obviously not specific for senescent cells. Therefore, we do not consider MTD as a senolytic agent.

The MTD concentration resulting in reduction of viability and induction of apoptosis was in the range of 10–100 µg/mL (significant apoptosis was induced in the most sensitive line LN229 at a concentration >20 µg/mL). This is far above the serum concentration that can be achieved in patients. Thus, the clinical tolerable serum level in humans was reported to be between 0.3 and 1.3 µg/mL [23]. In most studies the plasma levels in maintenance patients were <1 µg/mL (for a compilation of published data see Table S2). Therefore, a cytotoxic MTD concentration sufficiently high enough to exert a therapeutic anticancer effect is unlikely to be achieved in a therapeutic setting. Conclusions in line with this were drawn from studies using a panel of glioblastoma cell lines expressing the µ-opioid receptor. The authors found no interaction of MTD with TMZ [24]. Similar observations were made in an independent study with glioblastoma cells and MTD administered together with TMZ or IR (4 Gy) [25]. It should be noted that in this study TMZ was used at high dose of 200 µM, which causes mainly cytotoxic N-alkylations in the DNA. In our cytotoxicity studies, we used TMZ at lower doses (25 and 50 µM). Although under these conditions apoptosis is triggered exclusively by *O*^6^-methylguanine [26], we also observed that MTD and TMZ do not act synergistically, supporting the notion that MTD is not useful for enhancing the anticancer effect of TMZ.

It is important to note that the TMZ serum concentration in a therapeutic setting with a single dose of 150 mg/m^2^ is in the range of 20–30 µM [27] and following oral 200 mg/m^2^ TMZ, a plasma peak level of 72 µM and a cerebrospinal fluid level of 9.9 µM were determined [28]. This is close to the TMZ concentration that induces cell death in vitro (this study and ref. [21] where it is shown that apoptosis increases, without a threshold, linearly with dose of TMZ in LN229 cells). Moreover, TMZ in GBM therapy is administered repeatedly with daily doses of 50–130 mg/m^2^ or even higher [29,30]. This causes accumulation of toxic *O*^6^-methylguanine adducts in tumors not able to repair the lesion (MGMT promoter methylated) and, therefore, cytotoxic effects brought about by cumulative doses should be taken into account. In contrast to this, methadone as a non-genotoxic agent has likely a threshold for eliciting cytotoxicity (see dose-response curves). However, if methadone accumulates in the tumor, the critical target concentration might be higher than anticipated from the plasma level. Whether, under these conditions, toxic concentrations can be achieved without side effects on the normal tissue is, however, doubtful. It would be interesting to determine the tumor-MTD concentration and to elucidate whether locoregional administration of MTD elicits a tumor-specific effect.

The usefulness of MTD as an adjuvant in cancer therapy has been addressed recently in studies with malignant melanoma. Moreover, in this case, no interaction was found between MTD and TMZ (as well as cisplatin) and no benefit was observed following concomitant treatment in a single-case report [31]. Although these studies cannot exclude the possibility that there are subgroups of tumors that are highly sensitive to MTD and, therefore, respond at MTD concentrations that are achievable in patients, including addicted cases tolerating dose escalation, the data obtained so far are not encouraging, leading to the conclusion that MTD cannot be recommended as monotherapy or adjuvant radio-chemotherapy of gliomas.

## 4. Material and Methods

### 4.1. Cell Lines and Culture Conditions

The human glioblastoma cell lines LN-229 and A172 were purchased from American Type Culture Collection (ATCC). LN-229-MGMTc.12 was generated by stable transfection of LN-229 with a human MGMT cDNA expression vector and described previously [11,20]. VH10T were a kind gift of Prof. L. Mullenders, Leiden. Cells were cultured in DMEM with GlutaMAX (Gibco, Life Technologies Corporation, Paisley, UK) and 10% fetal calf serum. Cells were maintained at 37 °C in a humidified 6% CO_2_ atmosphere. Cells were seeded 24 up to 48 h before treatment when they reached exponential growth. For short term experiments (harvest 3 days after treatment), 2 × 10^5^ cells were seeded per 5-cm or 6-well dish (in 5 or 4 mL medium, respectively), for long-term experiments (apoptosis and senescence following TMZ) 5 × 10^4^ cells were seeded per dish. Under these conditions, cells were kept in exponential growth for the whole experimental period.

### 4.2. Drugs and Drug Treatment

d,l-methadone (MTD) and naloxone were purchased from Sigma-Aldrich (Taufkirchen, Germany). Temozolomide (TMZ) was obtained from Dr. Geoff Margison (University of Manchester, Manchester, UK). MTD and naloxone were dissolved in phosphate buffered saline (PBS; stock solution 5 mg/mL) and stored in batches at −20 °C. TMZ was dissolved in DMSO (150 mM) and stored in 50 µL batches at −80 °C until use. Immediately before treatment, it was diluted 1:10 in sterile distilled water and added to the cell culture medium at the desired final concentration. The amount of DMSO in the medium did not exceed 0.05% and was without any toxic effect (controls). Naloxone was added 30 min before MTD was added to the medium.

### 4.3. Western Blotting and MS-PCR

Protein extracts and immunoblotting occurred according to standard procedures as described previously [19]. Antibodies used were Anti-MOR1 D-12 (1:1000, Santa Cruz Biotechnology, Heidelberg, Germany) combined with goat anti-mouse IgG-HRP (1:5000, Santa Cruz Biotechnology) and HSP90 (Santa Cruz Biotechnology) as loading control. Immunoblots were developed with enhanced chemiluminescence (Amersham Biosciences, Freiburg, Germany).

For methylation-specific PCR (MS-PCR), 2 × 10^5^ cells were seeded and treated 2 days later with MTD at a subtoxic concentration (10 µg/mL). Cells were harvested by trypsinization 72 h later and subjected to MS-PCR, which occurred essentially as described previously [32].

### 4.4. Viability Assay

The MTT assays was performed according to the manufacturer’s instructions. MTT reagent (thiazolyl blue tetrazolium bromide) was obtained from BIOMOL (Hamburg, Germany).

### 4.5. Quantification of Apoptosis and Necrosis

The fraction of apoptotic and necrotic cells was determined using annexin V and propidium iodine (A/PI) staining of cells and measured by flow cytometry. For analysis, trypsinized cells including the supernatant were collected, transferred into 15 mL falcon tubes, washed with PBS and stored on ice. For labeling, cells were incubated for 15 min at RT in 50 µL 1× annexin binding buffer containing 2.5 µL annexin V/FITC (Miltenyi Biotec GmbH, Bergisch Gladbach, Germany) on ice. For PI staining, 10 µL PI from a 1 mg/mL stock solution (Sigma-Aldrich, Steinheim, Germany) and annexin binding buffer were added to each sample. Cells were incubated for additional 10 min. Cells were kept in the dark until measurement. Data acquisition was performed using the FACS Canto II flow cytometer (Becton Dickinson GmbH, Heidelberg, Germany) and the data was analyzed using the BD FACSDiva software. Apoptotic cells were defined as annexin V^+^/PI^−^ cells, whereas necrotic cells were defined as annexin V^+^/PI^+^ cells (see Figure S2). Experiments were performed in duplicate and repeated at least twice.

### 4.6. Quantification of Cellular Senescence

Senescence was determined by the amount of SA-β-galactosidase within the cells. To inhibit endogenous β-galactosidase activity, cells were pre-incubated with 300 µM chloroquine for 30 min in the incubator after which C12FDG was added to each sample to a final concentration of 33 µM. Following a 90 min incubation period in the incubator, cells were washed once with cold PBS and collected by trypsinization. Cell pellets were washed once with cold PBS, then resuspended in an adjusted amount of cold PBS and stored on ice. Cell were kept in the dark from the addition of c12FDG to the measurement. Data acquisition was performed using the FACS Canto II flow cytometer (Becton Dickinson GmbH, Heidelberg, Germany) and the data was analyzed using the Flowing Software 2 program (Perttu Terho, Turku Center for Biotechnology, University of Turku, Finland). Untreated, proliferating cells were measured, and the gate was set accordingly. Cells with a fluorescence higher than the control were defined as senescent.

### 4.7. Quantification of γH2AX Foci

The γH2AX foci assay we conducted essentially as described in detail before [21]. Evaluation of γH2AX stained nuclei of cells grown on coverslips occurred by laser scanning microscopy (LSM). At least 75 cells were assessed per experiment and experiments were performed twice.

### 4.8. Comet Assay

Cells were seeded in 6-well plates and treated 2 days later with the drugs. Moreover, 24 h thereafter, cells were harvested by trypsinization and embedded in low-melting point agarose. The alkaline comet assay was essentially performed as previously described [33]. The experiments were repeated twice.

### 4.9. Statistics

Data are presented as the mean of at least three independent experiments, ± SEM, and were treated statistically by the unpaired t-test and the two-way ANOVA test.

## Supplementary Materials

Supplementary Materials can be found at https://www.mdpi.com/1422-0067/21/19/7006/s1.
