# Supplementary material for: Cytotoxic and Senolytic Effects of Methadone in Combination with Temozolomide in Glioblastoma Cells
Source: Int J Mol Sci. 2020 Sep 23;21(19):7006. doi: 10.3390/ijms21197006 (PMC7582495; doi:10.3390/ijms21197006)
Supplement: Supplementary file 1 [file ijms-21-07006-s001.pdf]

Cytotoxic and senolytic effects of methadone in combination with temozolomide in glioblastoma cells

Bernd Kaina, Lea Beltzig, Andrea Piee-Staffa, Bodo Haas

Supplementary material

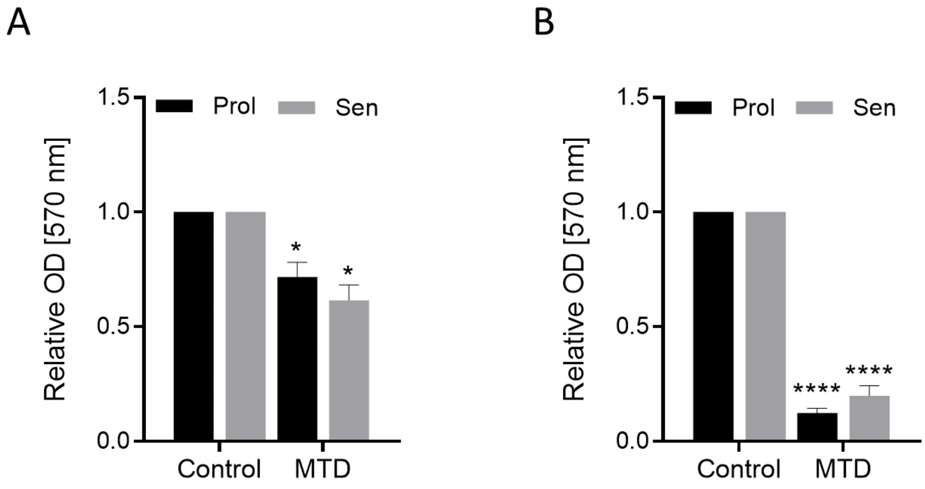

Figure S1
Viability of LN229 (A) and A172 (B) cells that were in the proliferating (Prol) or senescent (Sen) state when treated with methadone (40 µg/mL). Cellular senescence was achieved by TMZ treatment (50 µM, 8 days post-incubation at 37°C). Proliferating and senescent cells were reseeded for MTT assay. Two days after addition of MTD to the medium (96-well dishes) cells were processed for MTT staining according to the manufacturer‘s protocol and measured at OD 570. Data are set in relation to the corresponding non-treated controls. Each measure points represents the mean of quadruplicates. * $p<0.05$; **** $p<0.001$

Supplementary material

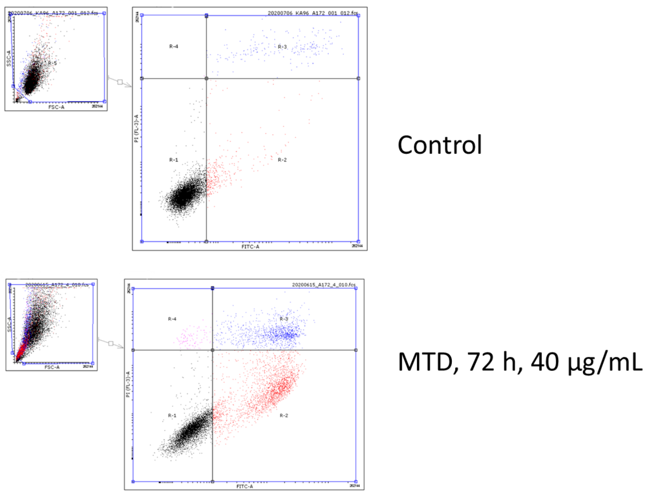

Figure S2
Flow cytometry of A172 cells not treated (control) and treated with methadone.
Ordinate: propidium iodide staining intensity, abscissa: annexin V staining intensity;
Black, life cells; red, apoptotic population; blue, necrotic population.

Supplementary material

A

	Apotosis						Necrosis					
Control	5.1	2.98	5.43	6.06	4.34	2.58	3.7	2.34	3.54	2.35	1.56	1.54
TMZ 25 µM	11.63	10.08	17.11	18.94	13.41	12.89	4.0	4.34	5.09	4.1	4.36	3.63
5 µg/mL MTD	4.67	2.83	6.04	5.37	4.23	2.43	1.96	1.5	2.05	1.84	1.84	1.68
20 µg/mL MTD	9.7	13.48	6.94	7.37	4.74	4.99	2.53	3.08	3.95	3.02	2.49	2.36
TMZ 25 µM + 5 µg/mL MTD	12.29	12.06	12.55	17.22	12.99	14.31	4.85	4.86	5.28	4.83	3.11	3.75
TMZ 25 µM + 20 µg/mL MTD	13.7	20.61	18.05	23.48	18.12	14.64	5.71	5.33	5.63	5.67	4.89	4.48

	Expected induced cell death						Observed induced cell death					
TMZ 25 µM + 5 µg/mL MTD	4.66	8.11	12.35	13.43	12.04	12.39	8.34	11.6	8.86	13.64	10.2	13.94
TMZ 25 µM + 20 µg/mL MTD	10.26	20.34	15.15	16.61	13.2	15.63	10.61	20.62	14.71	20.74	17.11	15.0

B

	Apotosis						Necrosis					
Control	6.01	4.97	6.04	4.96	4.88	4.58	2.09	2.27	3.45	3.59	3.06	2.1
TMZ 25 µM	7.66	8.13	22.6	12.07	6.77	9.86	5.14	4.62	5.53	7.6	5.37	3.81
5 µg/mL MTD	7.19	5.74	6.21	5.66	5.9	2.93	2.48	2.38	3.77	3.76	3.47	2.71
20 µg/mL MTD	10.09	9.21	16.58	16.87	40.83	38.71	3.53	3.77	5.46	5.95	13.53	11.81
TMZ 25 µM + 5 µg/mL MTD	9.08	9.03	22.69	15.62	7.43	7.97	4.42	4.95	4.6	6.24	5.25	4.37
TMZ 25 µM + 20 µg/mL MTD	16.09	15.94	27.5	19.43	46.87	53.1	7.6	7.45	8.3	8.25	15.05	14.43

	Expected induced cell death						Observed induced cell death					
TMZ 25 µM + 5 µg/mL MTD	6.46	7.37	19.19	10.21	7.09	6.35	6.46	6.39	19.33	11.81	5.2	5.58
TMZ 25 µM + 20 µg/mL MTD	45.14	12.61	18.97	20.85	45.24	51.53	58.6	16.48	26.64	19.32	46.65	60.51

Table S1
Apoptosis and necrosis frequencies in cells treated with TMZ and MTD. Expected induced cell death: Sum of induced apoptosis and necrosis measured after singe agent treatment. Observed induced cell death: Induced apoptosis and necrosis measured in the population concomitantly treated with TMZ and MTD. Induced frequencies: controls (untreated population) were subtracted from the treated population.
A, LN229; B, A172.

Supplementary material

Reference	Dose	Route	Plasma level	Patients
Dole et al, 1973	100 mg/day	oral	0.58 - 0.91 μg/mL	Maintenance patients
Foster et al, 2000	7.5 – 130 mg/day	oral	L-methadone: 0.251 μg/mL<br>D-methadone: 0.303 μg/mL	Maintenance patients
Inturrisi et al, 1987	10 – 30 mg	IV	0.3 – 1.3 μg/mL	Chronic use, pain patients
Lehotay et al, 2005	20 – 205 mg/day	oral	L-methadone: 0.06 – 2.84 μM (0.018 - 0.85 μg/mL)<br>D-methadone: 0.12 – 2.04 μM (0.036 – 0.61 μg/mL)	Maintenance patients
Linares et al, 2015	10 mg single dose	oral	up to ~0.04 μg/mL	Methadone treatment naïve patients
Mohamad et al, 2013	30 – 160 mg/day	oral	0.03 – 0.7 μg/mL	Maintenance patients
Wolff et al, 1993	10 – 60 mg/day	oral	0.13 – 0.68 nM<br>(0.04 - 0.2 ng/mL)	Opioid addicts

Dole VP, Kreek MJ: Methadone plasma level: sustained by a reservoir of drug in tissue. *Proc Natl Acad Sci U S A* 1973, 70(1):10.

Foster DJ, Somogyi AA, Dyer KR, White JM, Bochner F. Steady-state pharmacokinetics of (R)- and (S)-methadone in methadone maintenance patients. Br. J. Clin. Pharmacol. 50(5), 427–440 (2000).

Inturrisi, C.E., et al., Pharmacokinetics and pharmacodynamics of methadone in patients with chronic pain. Clin Pharmacol Ther, 1987. 41(4): p. 392-401.

Lehotay DC, George S, Etter ML, Graybiel K, Eichhorst JC, Fern B, Wildenboer W, Selby P, Kapur B. Free and bound enantiomers of methadone and its metabolite, EDDP in methadone maintenance treatment: relationship to dosage?, 2005, Clin Biochem 38:1088–1094.

Linares OA, Fudin J, Daly A, Schiesser WE, Boston RC: Methadone Recycling Sustains Drug Reservoir in Tissue. *J Pain Palliat Care Pharmacother* 2015, 29(3):261-271.

Mohamad N, Salehuddin RM, Ghazali B et al. Plasma methadone level monitoring in methadone maintenance therapy: a personalized methadone therapy. In: New Insights into Toxicity and Drug Testing. Gowder S (Ed.) InTech, Rijeka, Croatia (2013).

Wolff K, Hay AW, Raistrick D, Calvert R: Steady-state pharmacokinetics of methadone in opioid addicts. *Eur J Clin Pharmacol* 1993, 44(2):189-194

Table S2
